# MSPEDTI: Prediction of Drug–Target Interactions via Molecular Structure with Protein Evolutionary Information

**DOI:** 10.3390/biology11050740

**Published:** 2022-05-13

**Authors:** Lei Wang, Leon Wong, Zhan-Heng Chen, Jing Hu, Xiao-Fei Sun, Yang Li, Zhu-Hong You

**Affiliations:** 1 Big Data and Intelligent Computing Research Center, Guangxi Academy of Sciences, Nanning 530007, China; lghuang@gxas.cn; 2 College of Information Science and Engineering, Zaozhuang University, Zaozhuang 277160, China; hujing@uzz.edu.cn (J.H.); sxf@uzz.edu.cn (X.-F.S.); 3 Computer Science and Technology, Tongji University, Shanghai 200092, China; chenzhanheng17@mails.ucas.ac.cn; 4 School of Computer Science and Information Engineering, Hefei University of Technology, Hefei 230601, China; 2021010123@mail.hfut.edu.cn; 5 School of Computer Science, Northwestern Polytechnical University, Xi’an 710129, China

**Keywords:** deep learning, drug–target interactions, extreme learning machine, convolutional neural network

## Abstract

**Simple Summary:**

Drug discovery is the process of identifying potential new compounds through biological, chemical, and pharmacological means. Billions of dollars are spent each year on research aimed at discovering, designing, and developing new drugs for a wide range of diseases. However, the research and development of new drugs remain time-consuming and sometimes difficult to complete. With the development of new experimental techniques, huge amounts of data are generated at different stages of drug development. Biomedical research, especially in the field of drug discovery, is currently undergoing a major shift towards “big data” applications of artificial intelligence technologies. Therefore, a key challenge for future drug discovery research is the development of robust artificial-intelligence-based predictive tools for drug–target interactions (DTIs) that can study biomedical problems from multiple perspectives. In this study, a deep-learning-based prediction model for DTIs was designed by combining information on drug structure and protein evolution to provide theoretical support for drug research.

**Abstract:**

The key to new drug discovery and development is first and foremost the search for molecular targets of drugs, thus advancing drug discovery and drug repositioning. However, traditional drug–target interactions (DTIs) is a costly, lengthy, high-risk, and low-success-rate system project. Therefore, more and more pharmaceutical companies are trying to use computational technologies to screen existing drug molecules and mine new drugs, leading to accelerating new drug development. In the current study, we designed a deep learning computational model MSPEDTI based on **M**olecular **S**tructure and **P**rotein **E**volutionary to predict the potential **DTI**s. The model first fuses protein evolutionary information and drug structure information, then a deep learning convolutional neural network (CNN) to mine its hidden features, and finally accurately predicts the associated DTIs by extreme learning machine (ELM). In cross-validation experiments, MSPEDTI achieved 94.19%, 90.95%, 87.95%, and 86.11% prediction accuracy in the gold-standard datasets enzymes, ion channels, G-protein-coupled receptors (GPCRs), and nuclear receptors, respectively. MSPEDTI showed its competitive ability in ablation experiments and comparison with previous excellent methods. Additionally, 7 of 10 potential DTIs predicted by MSPEDTI were substantiated by the classical database. These excellent outcomes demonstrate the ability of MSPEDTI to provide reliable drug candidate targets and strongly facilitate the development of drug repositioning and drug development.

## 1. Introduction

Drug research is a global development problem. In the past few decades, the drug-targeted therapy strategy has achieved great success [1,2]. Finding specific drugs for targets is the focus of pharmaceutical research and development, which has made an indelible contribution to human health [3]. However, the rate of new drug development has been declining in recent years, and the cost of research and development has been rising [4]. The main reason for this is that the early screening of a large number of drug candidates in drug research still relies mainly on time-consuming and labor-intensive experimental methods, and the later discovery of unsatisfactory efficacy or toxic side effects of drugs leads to the failure of development. Therefore, efficient and high-throughput computational techniques in the early stages of drug research can play an important role in targeting and saving costs in early development [5,6,7,8].

With the rapid development of bioinformatics, many achievements have been achieved by using computational and simulation approaches to predict DTIs. Quantitative structure–activity relationship (QSAR) utilizes the physicochemical properties or structural parameters of the molecule to quantitatively study the interaction between small molecules and biological macromolecules by means of mathematics. Casañola-Marti et al. proposed a QSAR model for predicting anti-tyrosinase activity and demonstrated the effectiveness of the model in subsequent in vitro experiments, which greatly increased the rate of biochemical discovery of skin disease treatment [9]. Kar et al. proposed an approach to predict the carcinogenicity of drug compounds based on QSAR, which has been identified as a key factor in carcinogenicity by analyzing the contribution of molecular fragments to carcinogenicity [10]. Molecular docking (MD) is a computational simulation method for studying the optimal binding sites between drug molecules and target proteins by structural matching and energy matching and predicting their binding patterns and affinity [11]. Wallach et al. proposed a model to normalize docking scores through the virtually generated bait set that avoids the variability due to changes in physical properties when identifying active compounds in large screening libraries, thereby extending the applicability of the model [12].

Recently, computational methods for predicting DTIs based on protein target sequences have achieved excellent results and are favored by researchers for their use of reliable, high-quality characterization information enriched by raw data to ensure the accuracy of prediction results [13,14,15,16,17,18]. For instance, Lan et al. proposed a PUDT model combining protein target sequences and drug compound structures, which greatly improved the accuracy of DTI prediction using a weighted SVM classifier [19]. Cao et al. aimed to predict DTIs by using an extended structure–activity relationship method at the genome-scale level. In subsequent experiments, this approach gained good results [20].

In the present study, we combined protein sequence evolution with drug structure information to propose a deep learning MSPEDTI model to predict hidden DTIs. Concretely, MSPEDTI first fuses protein sequence information characterized by the Position-Specific Scoring Matrix (PSSM) and drug structure information characterized by molecular fingerprinting, and then automatically extracts them into continuous, low-dimensional, information-rich features using a deep learning CNN, thus avoiding the disadvantages of manual features such as tediousness, sparsity, and high dimensionality. Finally, the ELM classifier is used to accurately determine whether drug–target pairs are associated or not. In the gold-standard dataset, we evaluated MSPEDTI using the five-fold cross-validation (5CV) approach. Compared with other previous methods, MSPEDTI was able to learn valid biological characteristics for predicting DTIs and showed better performance. The robustness of MSPEDTI is also demonstrated by the experimental results of the case study, which can provide effective candidate targets for new drug research. The supporting data used in this study can be downloaded from https://github.com/look0012/MSPEDTI (accessed on 1 April 2022).

## 2. Materials and Methods

### 2.1. Gold-Standard Datasets

In the present study, we implemented the MSPEDTI model using the gold-standard datasets enzyme, GPCR, ion channel, and nuclear receptor, which were collated by Yamanishi et al. [21] from the BRENDA [22], KEGG [23,24], SuperTarget [25], and DrugBank [26] databases. After removing the redundant information, the numbers of DTI pairs contained in these datasets are 2926, 635, 1467, and 90, respectively. All of these pairs are constructed as positive datasets. Table 1 presents the statistical information for these gold-standard datasets.

The corresponding negative dataset construction process is as follows: firstly, all drug–target interaction pairs are divided into drug and target components; secondly, these drug and target are recombined into DTI pairs, and the pairs of interactions are removed. Finally, these drug–target pairs are randomly selected to construct the negative dataset, which is the same size as the positive dataset.

### 2.2. Drug Structure Characterization

We employed molecular fingerprints in this study to characterize the drug structures for the purpose of numerical conversion. The design idea of fingerprints is to characterize the molecular structure using the form of a dictionary collection of molecular fragments, which converts a drug molecule into a binary vector of values by determining whether certain fragments, i.e., molecular substructures, are present in the molecule. It first divides the molecular structure to obtain the structural fragments, and then encodes the fragments of these molecular structures into numbers according to certain rules and corresponds to each bit of the binary string, thus combining them as a whole (binary string) as a characterization of the molecular structure.

At present, the commonly used molecular fingerprints are FP4 fingerprint, MACCS fingerprint, Estate fingerprint, and PubChem fingerprint, and their corresponding molecular structure fragment numbers of 307, 166, 79, and 801. In this experiment, molecular fingerprints from the PubChem database were selected to characterize the drug structure of DTIs. The drug molecule is decomposed into 881 substructures in this descriptor. Given a drug, encode its corresponding bit as 1 or 0 depending on whether its molecular substructure is present. The fingerprint is encoded in Base64 on the PubChem website and provides a text description of it in binary, available for download from https://pubchem.ncbi.nlm.nih.gov/ (accessed on 1 January 2018).

### 2.3. Target Protein Characterization

In the experiments, the Position-Specific Scoring Matrix (PSSM) was used to numerically characterize the target protein. The PSSM can effectively describe the evolutionary information of protein amino acids, and it is commonly used in protein secondary structure prediction [27], protein binding site prediction [28], disordered region prediction [29], and distantly related protein detection [30,31] domains. The PSSM is a matrix of H×20, where H is the length of the protein, and 20 is the type of amino acid. The PSSM $Pssm=\left\{\Theta_{i,j}:i=1\cdots H\;and\;j=1\cdots 20\right\}$ can be expressed equationally as follows:

(1) $$Pssm=\left[\begin{array}{cccc} \Theta_{1,1} & \Theta_{1,2} & \cdots & \Theta_{1,20}\\ \Theta_{2,1} & \Theta_{2,2} & \cdots & \Theta_{2,20}\\ \vdots & \vdots & \vdots & \vdots \\ \Theta_{H,1} & \Theta_{H,2} & \cdots & \Theta_{H,20} \end{array}\right]$$

Here, the matrix element $\Theta_{i,j}$ indicates the probability that the i-th residue of the protein mutates to the i-type amino acid during the evolutionary process.

In the implementation, we utilized the Position-Specific Iterated BLAST (PSI-BLAST) [32] to calculate the PSSM by comparing it with the SwissProt database. We followed the previous study, setting the parameter iterations and e-value of the PSI-BLAST tool to 3 and 0.001 to obtain high homologous sequences in the experiment. The database and tool are available for download from http://blast.ncbi.nlm.nih.gov/Blast.cgi (accessed on 18 March 2002).

### 2.4. Feature Extraction

In the MSPEDTI model, the convolution neural network (CNN) algorithm of deep learning is used to extract the hidden features of the protein. Deep learning can learn the intrinsic patterns and levels of representation of sample data, thus enabling machines to have the same analytical learning capabilities as humans. As one of the representative algorithms of deep learning, CNN is able to classify the input information in a translation-invariant manner by hierarchical structure, thus deeply mining the essential features of data. Therefore, we introduced it into MSPEDTI to greatly strengthen the model prediction capability.

**Figure 1:**
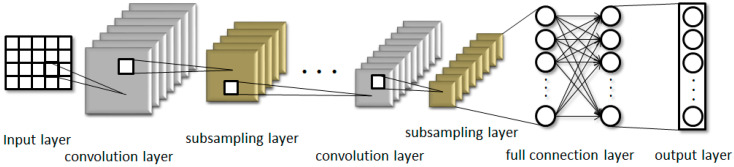
Schematic diagram of the structure of CNN.

CNN is a feedforward neural network with artificial neurons that respond to a portion of the surrounding units in the coverage area, including convolutional, pooling, sampling, fully connected, input, and output layers. With its special structure of local weight sharing, CNN has unique advantages in feature extraction, and its layout is closer to the actual biological neural network. CNN has unique superiority in feature extraction, with its special structure of local weight sharing, and its layout is closer to the actual biological neural network. Weight sharing reduces the complexity of the network, especially the feature that multidimensional input vectors can be directly input into the network, which avoids the complexity of data reconstruction in the process of feature extraction and classification. The structure diagram of CNN is shown in Figure 1. Assuming that $C_{i}$ is the feature map of layer  ith, its description can be:

(2) $$C_{i}=g\left(C_{i-1}\cdot W_{i}+b_{i}\right)$$

Here, operator · indicates convolution operations, $b_{i}$ indicates the offset vector, $W_{i}$ indicates the weight matrix of the ith layer convolution kernel, and g(x) indicates the activation function. The subsampling layer follows the convolutional layer and samples the feature map according to specific rules. Let $C_{i}$ be the subsampling layer with the following sampling rules:

(3) $$C_{i}=subsampling\left(C_{i-1}\right)$$

After multiple convolution and sampling, the features are classified by the fully connected layer to yield the data distribution Γ of the original input. Fundamentally, CNN can be regarded as a mathematical model that uses multilevel dimensional transformations to transform the original data $C_{0}$ into a new feature representation Γ.

(4) $$\Gamma \left(i\right)=Map(P=p_{i}|C_{0};\;\;\left(W,b\right))$$

Here, Γ represents the feature representation, $p_{i}$ indicates the ith label class, and $C_{0}$ represents the original data.

Minimizing the loss function H(W,b) is the ultimate goal of CNN training. Therefore, CNNs are typically trained to solve the overfitting problem by controlling the fitting strength using the parameter θ and adjusting the loss function L(W,b) by generalizing the norm.

(5) $$L\left(W,b\right)=H\left(W,b\right)+\frac{\theta }{2}W^{T}W$$

CNNs normally update their network layer parameters (W,b) layer by layer by gradient descent in the training phase and control the backpropagation function to exploit the learning rate ε.

(6) $$W_{i}=W_{i}-\varepsilon \frac{\partial E\left(W,b\right)}{\partial W_{i}}$$

(7) $$b_{i}=b_{i}-\varepsilon \frac{\partial E\left(W,b\right)}{\partial b_{i}}$$

### 2.5. Classification Prediction

The extreme learning machine (ELM) [33] is employed by MSPEDTI as a classifier to predict potentially associated DTIs. The ELM is a simple and effective single-hidden layer feedforward neural network learning algorithm that does not need to adjust the input weights of the network and the bias of the hidden elements during the execution and produces a unique optimal solution, so it has the advantages of fast learning and good generalization performance.

Given input samples $\left(X_{i},P_{i}\right)$ with L tagged, the ELM consisting of N neurons can be formulated as:

(8) $$\overset{N}{\underset{i=1}{\sum }}V_{i}g\left(W_{i}\cdot X_{j}+b_{i}\right)=O_{j},\;\;j=1,\ldots ,L$$

where $X_{i}={\left[x_{i1,}x_{i2,\ldots ,}x_{iL,}\right]}^{T}\in {\mathbb{R}}^{L}$, $P_{i}={\left[P_{i1},P_{i2},\ldots ,P_{im}\right]}^{T}\in {\mathbb{R}}^{m}$, g(x) indicates the activation function, $V_{i}$ indicates the output weight matrix, $W_{i}={\left[w_{i1},w_{i2},\ldots ,w_{iL}\right]}^{T}$ stands for the input weight matrix, $W_{i}\cdot X_{j}$ stands for the inner product of $W_{i}$ and $X_{j}$, and $b_{i}$ stands for the offset of the ith neurons.

To realize the minimization of the output error, i.e., the training goal of $\sum_{j=1}^{L}\|O_{j}-P_{j}\|=0$, the ELM needs to optimize its hyperparameters.

(9) $$\overset{N}{\underset{i=1}{\sum }}V_{i}g\left(W_{i}\cdot X_{j}+b_{i}\right)=P_{j},\;\;j=1,\ldots ,L$$

The equation can be simplified as follows:

(10) SV=P

(11) $$S=[{\begin{array}{ccc} g(W_{1}\cdot X_{1}+b_{1}) & \cdots & g\left(W_{N}\cdot X_{1}+b_{N}\right)\\ \vdots & \vdots & \vdots \\ g\left(W_{1}\cdot X_{L}+b_{1}\right) & \cdots & g(W_{N}\cdot X_{L}+b_{N}) \end{array}]}_{L\times N}\;\;V={\left[\begin{array}{c} V_{1}^{T}\\ \vdots \\ V_{N}^{T} \end{array}\right]}_{N\times m}\;\;P={\left[\begin{array}{c} P_{1}^{T}\\ \vdots \\ P_{L}^{T} \end{array}\right]}_{L\times m}$$

Here, V means the output weight, P means the expected output, and S means the hidden layer neurons output. To gain optimal performance, we want the ELM to acquire $\hat{W_{i}}$, $\hat{b_{i}}$ and $\hat{V_{i}}$, that is:

(12) $$\|S\left(\hat{W_{i}},\hat{b_{i}}\right)\hat{V_{i}}-P\|=\underset{W,b,V}{\min}\|S\left(W_{i},b_{i}\right)V_{i}-P\|\;\;\;\;i=1,2,\cdots ,N$$

This equates to minimizing the loss function

(13) $$E=\overset{L}{\underset{j=1}{\sum }}(\overset{N}{\underset{i=1}{\sum }}V_{i}g\left(W_{i}\cdot X_{j}+b_{i}\right)-P_{j}{)}^{2}$$

By the principle of the ELM algorithm, when the input weight $W_{i}$ and the offset $b_{i}$ of the hidden layer are ascertained, the ELM is able to uniquely obtain its output matrix. Therefore, the training problem of the ELM is transformed into the problem of solving the linear equation SV=P with a minimal and unique interpretation.

## 3. Results

### 3.1. Evaluation Indicators

We measured the performance of MSPEDTI in the present study using the evaluation indicators calculated by the five-fold cross-validation method (5CV). The 5CV approach first splits the whole dataset D into five subsets $D_{1},\ldots ,D_{5}$, which are roughly equal in size and do not intersect with each other. When testing subset $D_{i}$, the remaining subsets $D-D_{i}$ are fed into the classifier as the training set. Loop this operation until all subsets have been tested. The performance of MSPEDTI was evaluated by the average results and deviations of the five experiments. There are several evaluation indicators calculated through 5CV, which are described by the following equations.

(14) $$Accu.=\frac{TP+TN}{TP+TN+FP+FN}$$

(15) $$Sen.=\frac{TP}{TP+FN}$$

(16) $$Spec.=\frac{TN}{TN+FP}$$

(17) $$Prec.=\frac{TP}{TP+FP}$$

(18) $$MCC=\frac{TP\times TN-FP\times FN}{\sqrt{\left(TP+FP\right)\left(TP+FN\right)\left(TN+FP\right)\left(TN+FN\right)}}$$

where TP means true positive, TN means true negative, FP means false positive, and FN means false negative. Additionally, we plotted the operating characteristic curve (ROC) generated by 5CV and calculated its area under the curve (AUC) [34,35].

ROC is an essential metric for assessing the comprehensive performance of the model, which visualizes the variation between specificity and sensitivity and is displayed graphically. It computes a set of specificities and sensitivities by setting multiple different thresholds for successive variables, and then plots curves by using 1-specificity as abscissa and sensitivity as ordinate.

### 3.2. Assessment of Performance

**Figure 2:**
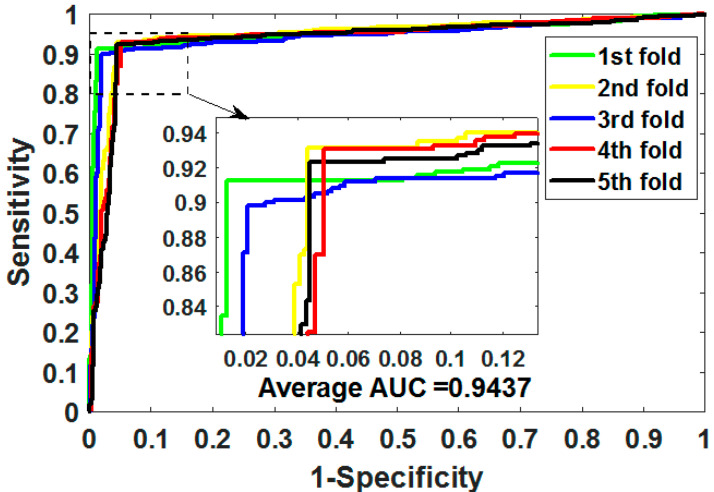
ROC of 5CV mapped by MSPEDTI on enzyme dataset.

**Figure 3:**
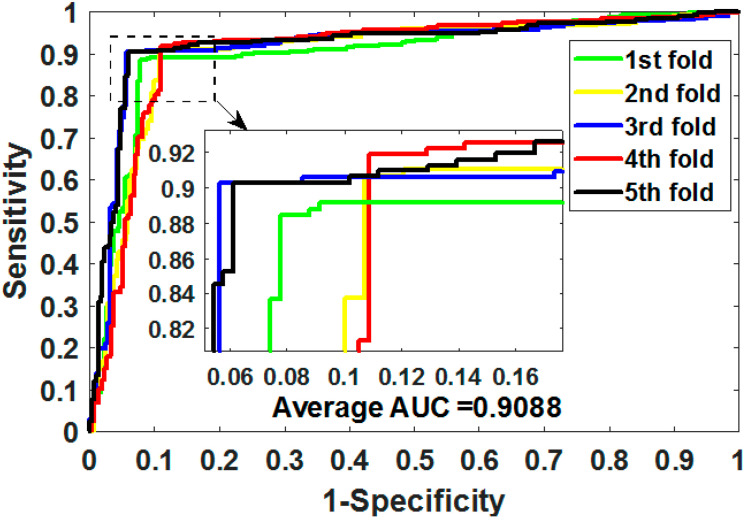
ROC of 5CV mapped by MSPEDTI on ion channel dataset.

**Figure 4:**
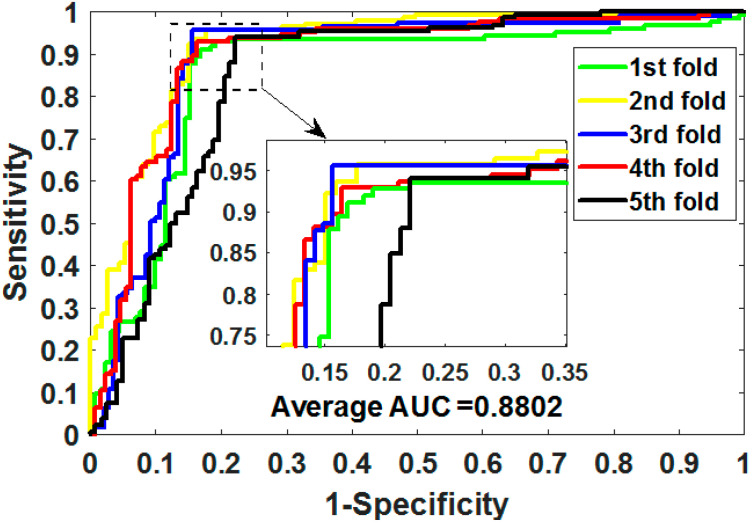
ROC of 5CV mapped by MSPEDTI on GPCR dataset.

**Figure 5:**
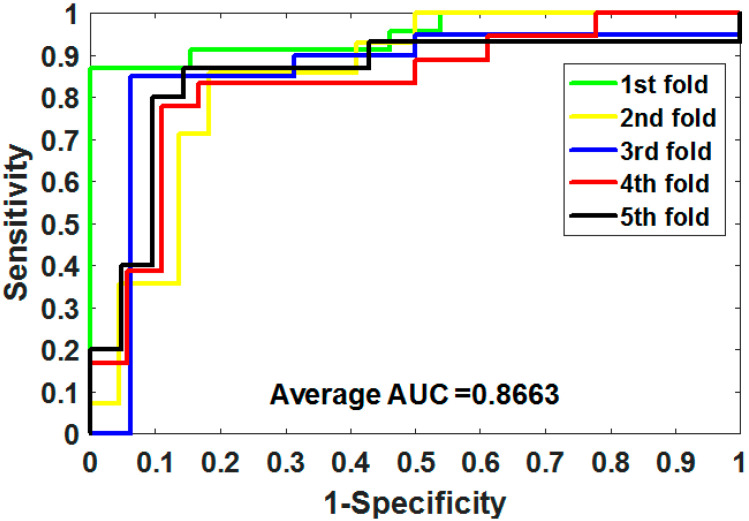
ROC of 5CV mapped by MSPEDTI on nuclear receptor dataset.

Gold-standard dataset enzymes, ion channels, GPCRs, and nuclear receptors were used to measure the capabilities of MSPEDTI in the experiment. The detailed outcomes of 5CV obtained by MSPEDTI on these datasets are listed in Table 2, Table 3, Table 4 and Table 5, respectively. From these tables, it is possible to observe that MSPEDTI accomplished satisfactory prediction accuracy, with values of 94.19%, 90.95%, 87.95%, and 86.11%, and their standard deviations were 0.41%, 1.10%, 1.51%, and 4.39%, respectively. In the enzyme dataset, the accuracy of all five MSPEDTI experiments was higher than 93.85%, with the highest result reaching 94.87%, and their standard deviations values were 94.87%, 94.27%, 93.85%, 94.02%, and 93.94%, respectively. MSPEDTI achieved good results of 88.51%, 81.95%, 76.41%, and 72.46% on MCC, which was used to measure classification performance, and its standard deviations were 0.89%, 2.24%, 2.88%, and 8.97%, respectively. On the comprehensive performance assessment index AUC, MSPEDTI gained 94.37%, 90.88%, 88.02%, and 86.63%, with standard deviations of 0.59%, 0.97%, 2.88%, and 4.77%, respectively. Additionally, MSPEDTI also yielded more satisfactory outcomes in terms of sensitivity and precision. The ROC curves produced by MSPEDTI for 5CV on the four gold-standard datasets are shown in Figure 2, Figure 3, Figure 4 and Figure 5.

### 3.3. Comparison of Different Descriptor Model

To estimate the impact of feature descriptors on MSPEDTI performance, we compared it with the two-dimensional principal component analysis (2DPCA) descriptor model. 2DPCA is an advanced version of the principal component analysis algorithm [36], which does not need to convert raw data into one-dimensional vectors, which is equivalent to removing the correlation of the row vector or column vector of the matrix. So, it can directly calculate the covariance training sample matrix and has the advantage of calculating the feature vectors quickly.

**Figure 6:**
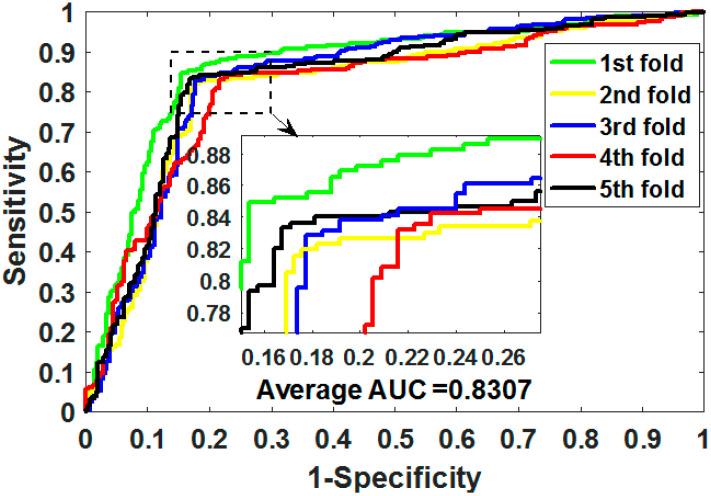
ROC curves plotted by the 2DPCA descriptor model on ion channel.

To validate the representation capability of the features extracted by CNN, we compared it with the 2DPCA descriptor on the ion channel dataset. In the interest of fairness, the other modules in MSPEDTI were kept unchanged, and only the feature extraction module was replaced. The 5CV results produced by the two descriptor models on the ion channel dataset are shown in Table 6, in which it can be observed that the MSPEDTI-generated results are higher than the 2DPCA descriptor model. The experimental outcomes of the contrast indicated that the CNN algorithm extracts the features better than the 2DPCA algorithm in our model. Figure 6 shows the ROC curve plotted on the ion channel by utilizing the 2DPCA descriptor method.

### 3.4. Comparison with Different Classifier Model

To validate whether the classifier helps to improve the performance of MSPEDTI, we compared it with the SVM classifier model in the same dataset. The learning strategy of SVM is to maximize the sample interval, thus converting it to the solution of the convex quadratic programming problem [37,38]. Similar to the ablation experiments for the descriptor model, in the comparisons of the classifier models, we only replaced the ELM classifier with the SVM classifier and left the other modules unchanged.

**Figure 7:**
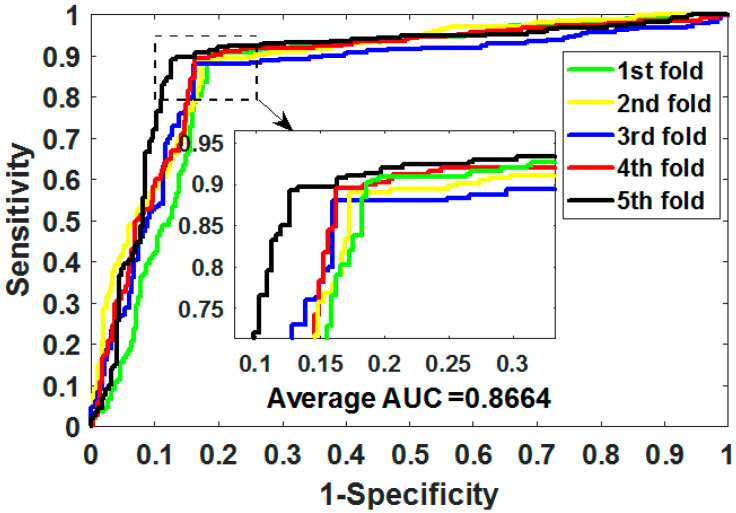
ROC curves plotted by the SVM classifier model on ion channel.

Table 7 presents the 5CV experimental outcomes of the MSPEDTI and SVM classifier model on the ion channel dataset. It is possible to observe from the table that the SVM classifier model performs well, and the accuracy, AUC, MCC, precision, and sensitivity are 86.48%, 86.64%, 73.05%, 83.86%, and 89.05%, respectively. However, compared with the ELM classifier, there are still some gaps, and the values of the above evaluation criteria are lower by 4.47%, 1.26%, 7.90%, 8.90%, and 4.24% respectively. These results indicate that the ELM classifier is indeed helpful to improve the prediction performance of MSPEDTI. Figure 7 shows the ROC curve plotted on the ion channel through utilizing the SVM classifier model.

### 3.5. Comparison with Previous Approaches

We compared MSPEDTI with previous methods in the gold-standard dataset to assess its ability to predict DTIs in a more intuitive way. Here, we picked the metric AUC, which best reflects the overall comprehensive capability of the model as the evaluation criterion. The AUC values resulting from these previous methods, including Yamanishi [4], DBSI [39], KBMF2K [40], Temerinac-Ott [41], NLCS [42], WNN-GIP [43], SIMCOMP [42], and NetCBP [44], are aggregated in Table 8. It can be observed from the table that MSPEDTI yielded optimal results in all four gold-standard datasets over the previous method. This suggests that the strategy of combining the CNN algorithm with the ELM classifier used by MSPEDTI can greatly enhance the ability to predict DTIs.

### 3.6. Case Studies

To further verify MSPEDTI’s ability in predicting new pairs, we trained it using all available data and predicted the unknown DTIs with the trained model. We searched the SuperTarget database [25] for the 10 highest-ranked DTI pairs of predicted associations. SuperTarget is a publicly available classic database that stores information about DTIs, and it currently collects 332,828 DTIs. Table 9 lists the top ten DTIs with the highest predictive score, from which we can see that seven potential DTIs were validated in the SuperTarget database. These outcomes indicated that MSPEDTI has outstanding capabilities in predicting new DTIs. Notably, while the rest of the three DTI interactions were not found in the current database, there is also the possibility of interaction between them.

## 4. Discussion

Accurate identification of the target protein of the drug can improve the efficacy of the drug and reduce side effects, thereby improving people’s health. In the current study, we presented a model MSPEDTI to predict DTI on the basis of protein evolution and molecular structures. The model takes full advantage of the protein evolutionary information and drug molecular information and uses a deep learning algorithm to mine the deep association between them. The experimental outcomes in the four gold-standard datasets revealed that the MSPEDTI model has outstanding performance.

However, there are still some shortcomings in our method: firstly, the number of DTIs known at present is still relatively small, and the model cannot be trained adequately; secondly, the parameters of the deep learning algorithm used in the model need to be further optimized to avoid overfitting in some cases; finally, how to integrate more biological information into the model is still worth further study.

## 5. Conclusions

In the present work, we designed a deep learning model MSPEDTI for predicting DTI on the basis of drug structure and protein evolution information. The model deeply excavates hidden features in protein evolutionary information by CNN, combines them with drug molecular fingerprint features, and uses ELM to efficiently predict potential DITs. The model on the gold-standard datasets enzymes, GPCRs, ion channels, and nuclear receptors, attained better 5CV results. To evaluate whether the modules used by MSPEDTI contribute to boost model performance, we implemented ablation experiments and compared them with other descriptor and classifier models. Furthermore, 7 of the 10 DTIs predicted by MSPEDTI were substantiated in authoritative databases. The exceptional results as mentioned above indicate that MSPEDTI has outstanding ability to predict DTIs and can provide reliable candidate targets for drug research. In the next step of our research, we will try to optimize the deep learning feature extraction method to mine more useful information from the raw data.

## Figures and Tables

**Table 1:** Statistical information for the four gold-standard datasets: the number of target proteins, drugs, and interaction pairs. Sparsity is the ratio of positive DTIs to all possible interactions.

Dataset	Target Proteins	Drugs	Interactions	Sparsity
Enzymes	664	445	2926	0.0099
Ion Channels	204	210	1467	0.0344
GPCRs	95	223	635	0.0299
Nuclear Receptors	26	54	90	0.0641

**Table 2:** MSPEDTI outcomes for 5CV on enzyme dataset.

Test Set	Accu. (%)	Sen. (%)	Prec. (%)	MCC (%)	AUC (%)
1	94.87	91.23	98.75	90.04	95.12
2	94.27	93.14	95.26	88.57	94.77
3	93.85	89.80	97.78	87.99	94.32
4	94.02	93.07	94.71	88.04	93.98
5	93.94	92.33	95.15	87.91	93.68
Average	**94.19 ± 0.41**	**91.91 ± 1.41**	**96.33 ± 1.81**	**88.51 ± 0.89**	**94.37 ± 0.59**

**Table 3:** MSPEDTI outcomes for 5CV on ion channel dataset.

Test Set	Accu. (%)	Sen. (%)	Prec. (%)	MCC (%)	AUC (%)
1	90.17	88.44	91.55	80.38	89.99
2	89.83	90.70	89.51	79.65	90.14
3	92.20	90.26	94.56	84.50	91.66
4	90.51	91.86	89.44	81.05	90.46
5	92.06	90.27	93.73	84.18	92.15
Average	**90.95 ± 1.10**	**90.31 ± 1.23**	**91.76 ± 2.36**	**81.95 ± 2.24**	**90.88 ± 0.97**

**Table 4:** MSPEDTI outcomes for 5CV on GPCR dataset.

Test Set	Accu. (%)	Sen. (%)	Prec. (%)	MCC (%)	AUC (%)
1	86.61	92.68	82.01	73.89	85.37
2	89.76	95.74	87.10	79.53	91.90
3	88.98	95.58	82.44	78.82	88.46
4	88.19	92.86	84.78	76.74	89.39
5	86.22	93.94	82.12	73.07	85.00
Average	**87.95 ± 1.51**	**94.16 ± 1.45**	**83.69 ± 2.22**	**76.41 ± 2.88**	**88.02 ± 2.88**

**Table 5:** MSPEDTI outcomes for 5CV on nuclear receptor dataset.

Test Set	Accu. (%)	Sen. (%)	Prec. (%)	MCC (%)	AUC (%)
1	91.67	86.96	100.00	84.05	94.98
2	80.56	85.71	70.59	61.51	84.74
3	88.89	85.00	94.44	78.26	85.63
4	83.33	83.33	83.33	66.67	83.02
5	86.11	86.67	81.25	71.81	84.76
Average	**86.11 ± 4.39**	**85.53 ± 1.45**	**85.92 ± 11.56**	**72.46 ± 8.97**	**86.63 ± 4.77**

**Table 6:** Comparison results of the 2DPCA descriptor model and MSPEDTI on ion channel.

Test Set	Accu. (%)	Sen. (%)	Prec. (%)	MCC (%)	AUC (%)
1	84.75	84.90	84.90	69.49	86.41
2	82.03	82.31	80.00	64.02	81.24
3	82.37	82.84	82.84	64.72	83.35
4	80.68	84.23	78.93	61.47	81.22
5	82.77	82.00	83.67	65.56	83.12
Average	**82.52 ± 1.47**	**83.26 ± 1.25**	**82.07 ± 2.52**	**65.05 ± 2.91**	**83.07 ± 2.12**
MSPEDTI	**90.95 ± 1.10**	**90.31 ± 1.23**	**91.76 ± 2.36**	**81.95 ± 2.24**	**90.88 ± 0.97**

**Table 7:** Comparison outcomes of SVM model and MSPEDTI on ion channel.

Test Set	Accu. (%)	Sen. (%)	Prec. (%)	MCC (%)	AUC (%)
1	85.76	90.14	81.42	71.81	85.08
2	85.93	89.04	82.70	71.94	87.90
3	85.76	87.34	84.04	71.46	84.80
4	86.61	89.49	83.73	73.34	87.10
5	88.34	89.26	87.41	76.70	88.33
Average	**86.48 ± 1.10**	**89.05 ± 1.04**	**83.86 ± 2.24**	**73.05 ± 2.16**	**86.64 ± 1.62**
MSPEDTI	**90.95 ± 1.10**	**90.31 ± 1.23**	**91.76 ± 2.36**	**81.95 ± 2.24**	**90.88 ± 0.97**

**Table 8:** Comparison of AUC with previous methods in the gold-standard dataset.

Method	Enzymes	Ion Channels	GPCRs	Nuclear Receptors
SIMCOMP	86.30	77.60	86.70	85.60
NLCS	83.70	75.30	85.30	81.50
Temerinac-Ott	83.20	79.90	85.70	82.40
Yamanishi	82.10	69.20	81.10	81.40
KBMF2K	83.20	79.90	85.70	82.40
WNN-GIP	86.10	77.50	87.20	83.90
DBSI	80.75	80.29	80.22	75.78
NetCBP	82.51	80.34	82.35	83.94
MSPEDTI	**94.37**	**90.88**	**88.02**	**86.63**

**Table 9:** Top 10 DTI pairs predicted by MSPEDTI.

Drug ID	Drug Name	Taregt Protein ID	Target Protein Name	Validation Source
D00951	Medroxyprogesteroneacetate	hsa2099	ESR1_HUMAN	SuperTarget
D00542	Bromochlorotrifluoroethane	hsa1571	CP2E1_HUMAN	SuperTarget
D03365	Transdermal Nicotine	hsa1137	ACHA4_HUMAN	SuperTarget
D00049	Nikotinsaeure	hsa 8843	G109B_HUMAN	SuperTarget
D00160	Epsilcapramine	hsa7298	TYSY_HUMAN	unconfirmed
D00771	Chlorzoxazone	hsa1374	CPT1A_HUMAN	unconfirmed
D00139	Xanthotoxine	hsa1543	CP1A1_HUMAN	SuperTarget
D00964	Letrozole	hsa1215	CMA1_HUMAN	unconfirmed
D00585	Mifepristone	hsa2099	ESR1_HUMAN	SuperTarget
D00437	Nifedipine Monohydrochloride	hsa1559	CP2C9_HUMAN	SuperTarget

## Data Availability

Not applicable.
